# Improvement of Locomotion Caused by *Lactococcus lactis* subsp. *lactis* in the Model Organism *Caenorhabditis elegans*

**DOI:** 10.3390/nu15204482

**Published:** 2023-10-23

**Authors:** Mohammad Shaokat Ali, Shamima Ahmed, Shino Takeuchi, Takayuki Wada, Eriko Kage-Nakadai

**Affiliations:** 1 Graduate School of Human Life Science, Osaka City University, 3-3-138, Sugimoto, Sumiyoshi-Ku, Osaka-shi 558-8585, Japan; shaokat@cvasu.ac.bd (M.S.A.); twada@omu.ac.jp (T.W.); 2 Faculty of Food Science and Technology, Chattogram Veterinary and Animal Sciences University, Khulshi, Chattogram 4225, Bangladesh; shamima@cvasu.ac.bd; 3 Graduate School of Human Life and Ecology, Osaka Metropolitan University, 3-3-138, Sugimoto, Sumiyoshi-Ku, Osaka-shi 558-8585, Japan; si22747f@st.omu.ac.jp; 4 Osaka International Research Center for Infectious Diseases, Osaka Metropolitan University, 1-2-7-601, Asahimachi, Abeno-Ku, Osaka-shi 545-0051, Japan

**Keywords:** *Caenorhabditis elegans*, Lactococcus, nisin, locomotion

## Abstract

*Lactococcus lactis* subsp. *lactis* exhibits probiotic properties in humans. Considering that *Caenorhabditis elegans* can be used to study the effects of microorganisms on animal behavior, owing to its simple nervous system, we assessed the impacts of two strains of *Lactococcus lactis* subsp. *Lactis*—a non-nisin-producing strain, NBRC 100933 (LL100933), and a nisin-producing strain, NBRC 12007 (LL12007)—on the lifespan, locomotion, reproductive capacity of, and lipid accumulation in, *C. elegans*. The lifespan of adult *C. elegans* fed a mixture (1:1) of *Escherichia coli* OP50 and LL100933 or LL12007 did not show a significant increase compared to that of the group fed a standard diet of *E. coli* OP50. However, the nematodes fed *Lactococcus* strains showed notable enhancement in their locomotion at all of the tested ages. Further, the beneficial effects of LL100933 and LL12007 were observed in the *daf-16* mutants, but not in the *skn-1* and *pmk-1* mutants. The lipid accumulation in the worms of the *Lactococcus*-fed group was lower than that in the control group at all experimental ages. Overall, LL100933 and LL12007 enhance the locomotor behavior of *C. elegans*, likely by modulating the PMK-1/p38 MAPK and SKN-1/Nrf2 transcription factors.

## 1. Introduction

Lactic acid bacteria have been recognized as a form of probiotics owing to their beneficial impact on host well-being [1]. Probiotics are live microorganisms that, when consumed, provide health benefits by improving or restoring the gut microbiota [2]. They are highly effective in treating human and animal diseases and have been widely used as microbial agents in livestock and poultry breeding [1]. *Lactococcus lactis* subsp. *lactis* is a gram-positive bacterium belonging to the genus *Lactococcus*; it is a subspecies of *Lactococcus lactis*, which contains four subspecies: *L. lactis* subsp. *lactis*, *L. lactis* subsp. *hordniae*, *L. lactis* subsp. *cremoris*, and *L. lactis* subsp. *tructae* [3]. *Lactococcus lactis* has demonstrated the ability to survive the harsh conditions of the gastrointestinal tract, maintain its viability in the intestine, and produce lactic acid, which helps to maintain an acidic environment in the gut and inhibits the growth of harmful bacteria [4]. *Lactococcus lactis* exhibits immunomodulatory properties by enhancing the production of cytokines and chemokines, which play essential roles in immune regulation. It also stimulates the production of immunoglobulin A (IgA), an important antibody involved in mucosal immunity [5]. Studies have shown that *Lactococcus lactis* possesses anti-inflammatory properties that aid the suppression of pro-inflammatory cytokines, thereby promoting gut homeostasis and serving as a preventive measure against chronic inflammatory conditions [6]. *Lactococcus lactis* has been reported to enhance the integrity of the gut barrier by strengthening the tight junctions between intestinal epithelial cells. This property reduces gut permeability and prevents the translocation of harmful substances into the bloodstream [7]. Bacteria produce antimicrobial compounds, such as bacteriocins, which prevent the growth of harmful bacteria. These antimicrobial properties play a vital role in maintaining a healthy and balanced gut microbiota [8].

*Lactococcus lactis* has gained attention because of its ability to produce nisin, an antimicrobial peptide with strong inhibitory effects against various foodborne pathogens [9]. Nisin, a 34-amino-acid peptide that exhibits broad-spectrum antimicrobial activity against gram positive bacteria, is recognized as the only bacteriocin permitted for use in its purified form, and has been assigned the European food additive number E234 [10]. The unique characteristics of nisin, including its high potency against important pathogenic and spoilage bacteria, low toxicity to humans, and rarity of bacterial resistance, have contributed to its effectiveness as a food preservative [10].

*Caenorhabditis elegans*, a bacterivorous nematode that exists in a free-living state, serves as a valuable experimental model because it can be easily cultivated and has a brief and consistent lifespan, as well as a wide array of genetic tools available for investigation [11]. At present, the *C. elegans* model is used worldwide for assessing probiotic strains [12,13]. *C. elegans* is commonly found in rich soil or compost as dauer, and is often associated with habitats containing rotten plant matter—especially rotten fruits, such as apples—and compost heaps. These environments provide a suitable niche for *C. elegans* because of the presence of microbes, which serve as a food source for the nematode and can colonize its gut [14]. *C. elegans* also provides a valuable platform for studying the effects of microorganisms on animal behavior, owing to its simple nervous system and well-characterized behavior. In a previous study, *C. elegans* was exposed to *Lactococcus cremoris* in order to understand how this probiotic bacterium influenced the neurobiological and behavioral responses of the nematode, and *Lactococcus cremoris* showed an improved lifespan and health span in *C. elegans* [15]. The effect of *Lactococcus lactis* subsp. *lactis* on the lifespan and movement of *C. elegans* is currently unknown. 

Therefore, we aimed to investigate how nisin-non-producing *Lactococcus lactis* subsp. *lactis* (LL100933) and nisin-producing *Lactococcus lactis* subsp. *lactis* (LL12007) affect the physiological functions of *C. elegans*, such as its lifespan, locomotion, and lipid accumulation.

## 2. Materials and Methods

### 2.1. Bacterial Strains and Culture Conditions

*Lactococcus lactis* subsp. *lactis* (NBRC 100933) and nisin-producing *Lactococcus lactis* subsp. *lactis* (NBRC 12007) were purchased from the NITE Biological Resource Centre (NBRC), Tokyo, Japan, and cultivated anaerobically in a trypticase soya yeast extract medium (814 medium) at 30 °C. These strains served as the nematode feed supplies. For nematode cultivation, *Escherichia coli* OP50 was utilized as the control feed supply, which was grown on tryptone soya agar (Nissui Pharmaceutical, Tokyo, Japan) at 37 °C. The bacteria cultured on the media were stripped using a sterile inoculation loop and collected in a microcentrifuge tube (Eppendorf, Hamburg, Germany) to weigh the culture. A suspension of gathered bacteria (100 mg wet weight) was prepared in 0.5 mL of M9 buffer (5 mM potassium phosphate, 1 mM CaCl_2_, and 1 mM MgSO_4_). For the experimental assays, 50 µL of the bacterial suspension was disseminated onto nematode growth media (modified as peptone-free, mNGM) plates (1.7% *w*/*v* agar, 50 mM NaCl, 1 mM CaCl_2_, 5 μg/mL cholesterol, 25 mM KH_2_PO_4_, and 1 mM MgSO_4_) (10 mg bacteria per plate). 

### 2.2. Nematodes and Growth Conditions

The Caenorhabditis Genetics Center, University of Minnesota, provided the wild-type *Caenorhabditis elegans* (Bristol N2) and its derivative mutant strains: CF1038 *daf-16* (*mu86*), outcrossed with N2 11 times by the Kenyon lab; VC1772 *skn-1* (*ok2315*/nT1[*qIs51*]), outcrossed once by the Moerman lab; and KU 25 *pmk-1* (*km25*), outcrossed six times by the Matsumoto lab. The nematodes were cultured and proliferated on nematode growth medium (NGM) using standard techniques [16]. The nematodes were cultured as follows. Eggs were produced by exposing mature *C. elegans* to a sodium hypochlorite/sodium hydroxide solution. To facilitate hatching and synchronization, the egg suspension was incubated in M9 buffer for one day at 25 °C. The resulting suspension of the synchronized L1-stage worms was centrifuged at 156× *g* for 1 min. After aspirating the supernatant, the remaining larvae were transferred onto mNGM plates coated with 10 mg of OP50. The transplanted worms were cultured at 25 °C for two days (referred to as three-day-old animals).

### 2.3. Lifespan Assay

For the lifespan assay, 70 synchronized three-day-old (young adult) nematodes were placed on two 5 cm mNGM dishes (35 animals per dish) coated with 10 mg *E. coli* OP50 alone or a mixture of 5 mg OP50 and LL100933 or LL12007. Following this, the plates were incubated at 25 °C. The nematodes were moved to fresh feed plates every day for the first four consecutive days, and were then moved every other day. The number of live and deceased nematodes was counted daily. A nematode was deemed deceased if it did not react to gentle touch with a worm picker. Nematodes that escaped from the plate or perished because of internal hatching were deemed lost and excluded from the study. All the assays were performed twice. The replicated statistics were combined to present the results.

The following formula was used to calculate mean lifespan (MLS) [17]:

$$MLS=\frac{1}{N}\sum_{j}\frac{X_{j}+X_{j+1}}{2}dj$$

where *N* is the overall number of nematodes and $d_{j}\;$ is the number of nematodes that died within the age range ($X_{j}$ to $X_{j+1}$). The following equation was used to determine the standard error (SE) of the estimated mean lifespan:

$$SE=\sqrt{\frac{1}{N(N-1)}\sum_{j}{\left(\frac{X_{j}+X_{j+1}}{2}-MLS\right)}^{2}d_{j}}$$

The maximum lifespan was determined as the average age of 15% of the nematodes in each group that lived the longest.

### 2.4. Locomotion Scoring of Nematodes

Young adult (three-day-old) nematodes were plated on mNGM plates with lawns of OP50, and a mixture (1:1) of OP50 and LL100933 or LL12007. The dishes were incubated at 25 °C. Nematode motility was then measured at various ages using a scoring technique, as described previously [18,19]. Briefly, nematodes were categorized as ‘class A’ when they moved vigorously or spontaneously in response to prodding, ‘class B’ when they moved incoherently or did not move unless prodded, and ‘class C’ when they only moved their heads and/or tails in response to prodding. The decreased nematodes that had died were categorized as ‘class D.’ The frequency of body bending in the worms was measured in S-basal media under a dissecting microscope, as this is intricately linked to their locomotion [20]. The locomotion assay was performed using two technical replicates for each sample. The combined data were statistically examined and presented.

### 2.5. Bacterial Selection Test

Three-day-old nematodes were placed on mNGM plates covered with OP50 lawns and mixtures of OP50, and either LL100933 or LL12007. The nematodes were then cultured until the late L4 stage. The nematodes were then subjected to a minimum of three washes with M9 buffer. In this assay, OP50 bacterial cultures were considered as controls (C), and (OP50 + LL100933) and (OP50 + LL12007) bacterial cultures were considered as tests (T). A non-seeded mNGM plate was divided into four equal segments, and OP50 cultures (suspended in M9 buffer), (OP50 + LL100933), or (OP50 + LL12007) were dropped onto opposite sections and equally spaced from the center. Approximately 100–200 nematodes fed OP50 and (OP50 + LL100933) or (OP50 + LL12007) were placed at the center of different plates, equidistant from the control and test cultures. Nematodes were allowed to move around freely for 1–2 h, and the percentage of nematodes that entered each bacterial lawn was calculated. Three independent replicates were performed for each group of nematodes fed a specific bacterial culture.

### 2.6. Brood Size

Two L4-stage hermaphrodites were transferred to an mNGM plate with lawns of OP50 and a combination of OP50 and either LL100933 or LL12007 to measure the nematode brood size. The assay used a total of 20 synchronized hermaphrodites that were moved daily to new mNGM feed plates until reproductive termination. The total number of offspring per plate was determined. Reproducibility was verified using ten independent replicates, and the combined results were statistically analyzed and presented.

### 2.7. Body Size

Three-day-old adult worms were introduced onto mNGM plates with lawns of OP50, LL100933, and LL12007, or a combination (1:1) of OP50 with LL100933 or LL12007. The plates were kept at 25 °C, and the size of the randomly selected live worms was measured every 24 h until they reached eight days of age [21]. Images of mature nematodes were captured and measured using a BZ-X800 microscope (KEYENCE, Tokyo, Japan) with the Hybrid Cell Count software (BZ-H3C).

### 2.8. Lipid Accumulation Staining

To investigate lipid accumulation in worms, we performed staining using a previously established method with slight modifications [22]. We examined lipid accumulation in worms of various experimental ages, including four-, five-, six-, and seven-day-old worms subjected to different feeding strategies (OP50, OP50 + LL100933, OP50 + LL12007, LL100933, and LL12007 feeding). Briefly, worms fed different feeds were washed thrice with M9 buffer. The specimens were immobilized in 50% isopropanol solution in phosphate-buffered saline (PBS) for 15 min on ice. The Oil Red O stock solution (0.5 g per 100 mL in isopropanol; Sigma-Aldrich, St. Louis, MO, USA) was diluted with distilled water (dH_2_O) to create a 60% working solution, which was then filtered through a 0.2 μm membrane filter. Immobile worms were placed in a functional solution and incubated at 25 °C for 20 min. After staining, immobilized worms were rinsed with M9 buffer containing 0.5% Triton X-100 and placed on glass slides for imaging. Worms were randomly selected for observation [23]. Imaging was performed using a BX53 microscope fitted with a DP73 color camera (Olympus, Tokyo, Japan). The dye intensity was then measured using the ImageJ software (v1.53), as described previously [24]. 

### 2.9. RNA Isolation and Sequencing

Worms in their adult stage (three days old) were cultivated for five days on mNGM plates coated with two different types of food as the control-fed group (OP50) and the test-fed groups (fed a mixture (1:1) of OP50 and LL100933 or LL12007). Approximately 200 worms were collected per group, subjected to at least three washes with M9 buffer containing 0.2% gelatin, and immersed in RNAlater solution (Qiagen, Hilden, Germany). The samples were stored at −80 °C until RNA extraction. Thawed nematode suspensions were pulverized using a microtube pestle (Scientific Specialties, Inc., Lodi, CA, USA) and blended with TRIzol (Thermo Fisher Scientific, Waltham, MA, USA). A RNeasy Lipid Tissue Kit (Qiagen) was used to isolate total RNA. Subsequently, RNA sequencing was performed by DNAFORM (Yokohama, Japan) as follows: mRNA purification was achieved using the Magnosphere™ UltraPure mRNA Purification Kit (Clontech, Mountain View, CA, USA). Subsequently, the SMARTer^®^ Stranded Total RNA-Seq Kit (Clontech) was used to create libraries, which were then subjected to HiSeq sequencing as 150 bp paired-end reads (Illumina Inc., San Diego, CA, USA). The RNA-seq outcomes were initially evaluated using FastQC (ver. 0.11.7) to gauge the quality. TrimGalore! (version 0.4.4), Trimmomatic (version 0.36) [25], and Cutadapt (version 1.16) were used for trimming and quality filtering the raw reads. The reads obtained after cleaning were aligned to the N2 *C. elegans* reference genome ce11 (WBcel235.91) using the STAR software (version 2.6.1a) [26]. Quantitative differential expression analysis was conducted between the control and test-fed groups using the featureCounts tool (version 1.6.1) [27] for the read counts of gene features, followed by DESeq (version 1.30.0) [28]. Enrichment analysis was performed on genes that exhibited a P.adjust value of <0.05 (Tables S1–S4). This analysis was conducted using clusterProfiler (version 3.6.0) to determine the enrichment of these genes in Gene Ontology (GO) categories, specifically for biological processes (BP), molecular functions (MF), and cellular components (CC) (Tables S5–S8).

### 2.10. Reverse Transcription and Quantitative Real-Time PCR

A QuantiTect Reverse Transcription Kit (Qiagen) was used to synthesize cDNA after removing genomic DNA. A StepOnePlus Real-Time PCR system (Thermo Fisher Scientific, Waltham, MA, USA) was used to conduct quantitative PCR (real-time PCR) using the FastStart Universal SYBR Green Master (ROX) (Roche Diagnostics, Mannheim, Germany). The PCR procedure involved the following settings: an initial denaturation step at 95 °C for 10 min, followed by 40 cycles of denaturation at 95 °C for 15 s, and annealing/extension at 60 °C for 1 min. The experiment included three biological replicates, and the relative mRNA expression was determined using the cycle threshold (ΔΔC_T_) method [29]. Expression was normalized to that of the housekeeping genes *act-1*, *tba-1*, and *cyc-1*. The primers used for real-time PCR are listed in Table S9.

### 2.11. Statistical Analysis

The Kaplan–Meier method was used to analyze the survival of *C. elegans*, while the Mantel–Cox log-rank test was employed to assess the statistical significance of both the survival curves and locomotion scores (GraphPad Prism 9.0). Differences in the brood size were examined using the Mann-Whitney U test. The body areas of nematodes with varying feeding habits were analyzed using the Two-Factor Factorial ANOVA and Tukey’s multiple comparison tests. The statistical significance of differences in the red dye intensity (Oil Red O stain), bacterial preference in the food choice assay, and relative gene expression were assessed using Student’s *t*-test. The data are presented as means with standard error of the mean (SEM). Statistical significance was set at *p* < 0.05.

## 3. Results

### 3.1. Lifespan and Locomotion of Wild-Type (N2) C. elegans

**Figure 1:**
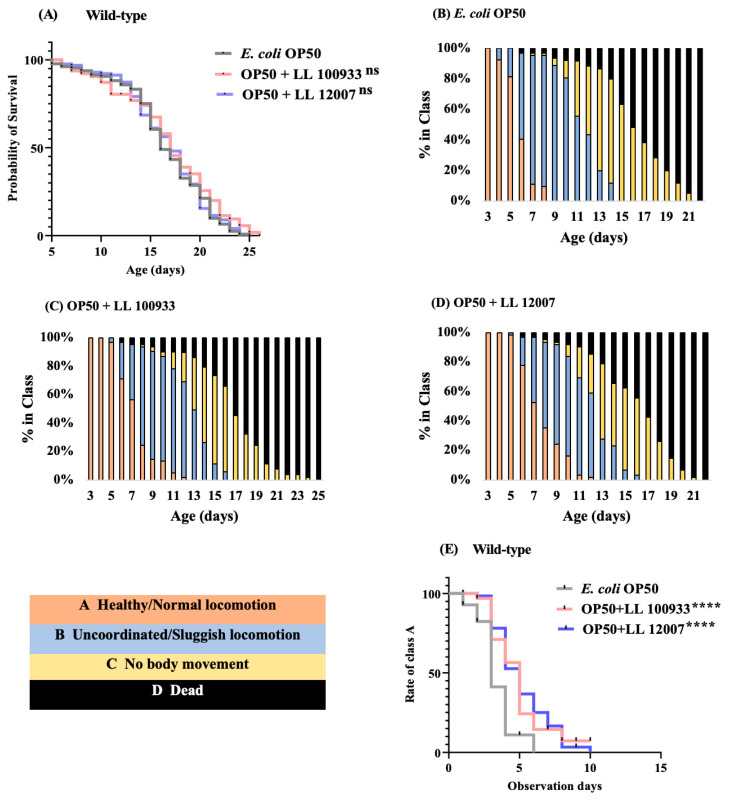
Lifespan assay of *C. elegans* with *Lactococcus lactis subsp. lactis* NBRC 100933 (LL 100933) and NBRC 12007 (LL 12007). (**A**) Survival curves of OP50 + LL 100933-fed and OP50 + LL 12007-fed wild-type *C. elegans* compared with those of the *E. coli* OP50-fed control worms. Age 0 nominal days was used to designate young adults. ns = Not significant, *p* > 0.05 vs. the control-fed worms. Asterisks indicate statistically significant differences (****, *p* < 0.0001) compared to the control worms fed *E. coli* OP50 using the log-rank (Mantel–Cox) test. Supplementary lifespan data and analysis are provided in supplementary Table S10. Age-related locomotor activity of *C. elegans* (wild type). (**B**–**D**) show the locomotory scoring scheme for worms fed OP50, OP50 + LL 10093, and OP50 + LL 12007. Worms were categorized into four classes as follows: Class A, Healthy/Normal locomotion (pink bars); Class B, Uncoordinated/Sluggish locomotion (blue bars); Class C, No body movement but head movement in response to prodding (yellow bars); and Class D, Dead worms (black bars). (**E**) Health span curves of wild type (N2) *C. elegans* fed OP50 + LL 100933 and OP50 + LL 12007 compared with those of OP50-fed control worms.

Initially, we examined the effect of dietary supplementation with *Lactococcus* strains (LL100933 and LL12007) on the longevity of *C. elegans*. As the worms avoided bacterial lawns of pure *Lactococcus* strains, they were fed a mixture of OP50 and LL100933 or LL12007. Consequently, wild-type worms nourished with *Lactococcus* strains exhibited the same lifespan as those fed with *E. coli* OP50 (Figure 1A). 

**Figure 2:**
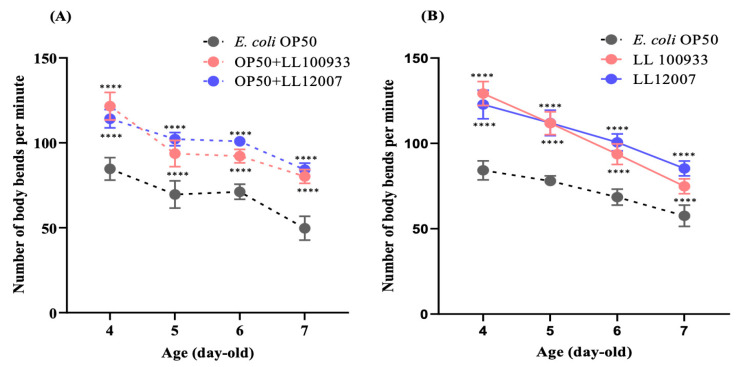
Body bending frequency (bends/minute) of (**A**) mixed-fed (OP50 + LL100933 and OP50 + LL12007) and (**B**) sole-fed (LL100933 and LL12007) worms compared with that of control-fed (OP50) worms. Worms fed with mixed and sole cultures of test strains had a significantly (*p* ≤ 0.0001) higher body bending frequency compared with that of control-fed worms. All data are expressed as means ± standard error. Asterisks (****, *p* ≤ 0.0001) indicate statistically significant differences compared to those of the control-fed group. Two-factor factorial ANOVA and Tukey’s multiple comparison tests were used to analyze the data where *n* = 20 worms.

Further, we assessed whether *Lactococcus* strains mitigated the age-related reduction in *C. elegans* locomotion. The locomotion scores of the worms fed *Lactococcus* strains were consistently higher than those of the worms fed OP50 across all of the examined ages (Figure 1B–D). The group fed a mixed culture of OP50 and LL100933 or LL12007 showed a notable increase in the proportion of worms in class “A”, which exhibited automatic movement or strong motion upon prodding (Figure 1E). The body bending rate is also considered to be closely correlated with locomotion. Therefore, a study was conducted to explore whether the ingestion of LL 1009333 and LL12007 led to an enhancement in the body-bending capability of worms. The frequency of body bending in the worms fed LL100933 or LL12007 was higher than that in the worms fed OP50 (Figure 2). 

### 3.2. Impacts of Lactococcus Feeding in Mutants

**Figure 3:**
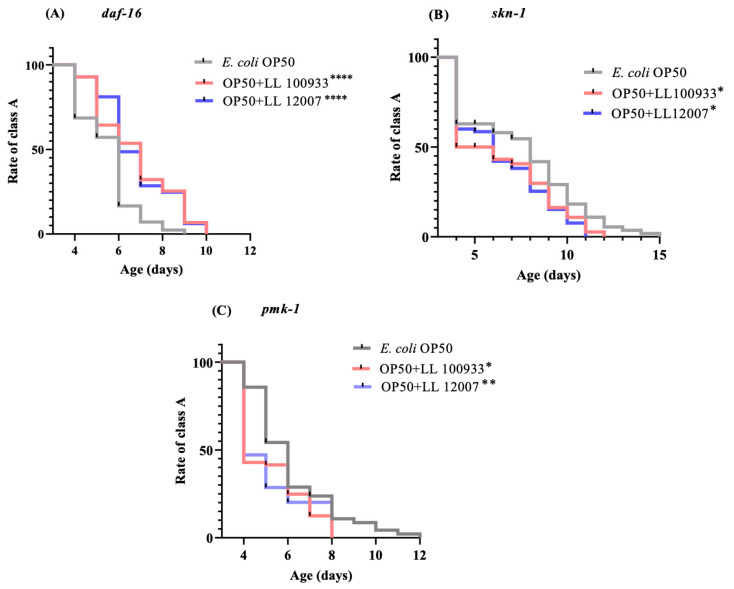
(**A**) Locomotion score of class A in mutants *daf-16* (*mu86*); (**B**) *skn-1* (*ok2315*); and (**C**) *pmk-1* (*km25*) fed with OP50 + LL 100933 and OP50 + LL 12007 compared with that of the control (OP50)-fed worms. Using the log-rank (Mante-Cox) test, the percentages of the frequency of Class A worms were examined. Asterisks (****, *p* < 0.0001; **, *p* ≤ 0.005; and *, *p* ≤ 0.05) indicate statistically significant differences between the test and control groups.

This investigation focused on mutants that lacked defense-related signaling pathways to explore the mechanisms responsible for the positive effects of *Lactococcus* strains on the locomotory behavior of *C. elegans*. The mechanism underlying the improved locomotion of nematodes due to *Lactococcus* was investigated by examining the loss-of-function mutants *daf-16*, *skn-1*, and *pmk-1*. We hypothesized that the advantageous effects of *Lactococcus* strains are mediated by *skn-1* and *pmk-1*. An improved locomotion score, i.e., rate of class A locomotion, was observed in the loss-of-function mutant *daf-16* (Figure 3A); however, decreased motion was observed in the loss-of-function mutants *skn-1* (Figure 3B) and *pmk-1* (Figure 3C). This outcome suggests that locomotion behavior in worms is boosted by regulating the p38 MAPK and Nrf2 transcription factors.

### 3.3. Body and Brood Sizes

**Figure 4:**
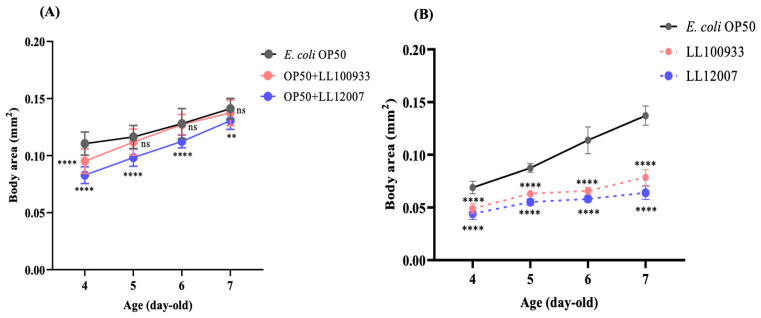
Growth of (**A**) control-fed (OP50) and mixed-fed (OP50 + LL100933 and OP50 + LL12007) and (**B**) control-fed (OP50) and sole test-fed (LL 100933 or LL 12007) worms from 4 to 7 days of age. All results are expressed as means ± standard error. On all observed days, the sole test-fed worms (LL 100933 or LL 12007) showed significantly decreased body size compared to that of control (OP50) fed worms. Asterisks indicate (****, *p* < 0.0001); **, *p* < 0.01) statistically significant differences between test-fed and control-fed groups. Two-factor factorial ANOVA and Tukey’s multiple comparison tests were used to analyze the data where *n* = 20 worms.

**Figure 5:**
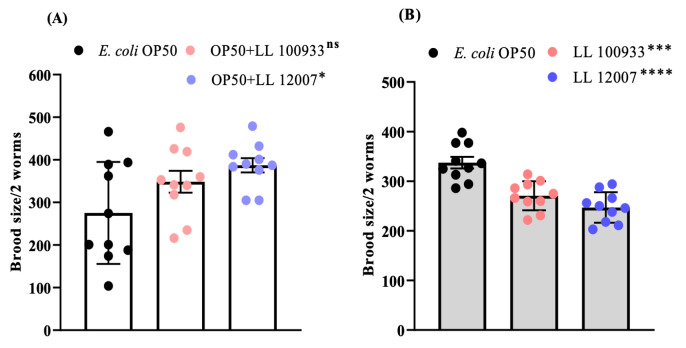
Comparative investigation of brood size among *C. elegans* N2. (**A**) Worms fed with OP50 + LL100933 (*n* = 20), and OP50 + LL12007 (*n* = 20) compared with OP50-fed (control) worms (*n* = 20). (**B**) LL100933-fed (*n* = 20) and LL12007-fed worms (*n* = 20) compared with control (OP50-fed) worms. Asterisks indicate statistically significant differences (*, *p* ≤ 0.05; ***, *p* ≤ 0.0004; ****, *p* < 0.0001) from the control (OP50-fed) worms. Data are shown as means ± standard error. Statistical analysis was carried out using the Mann-Whitney U test.

To test the possibility that the improved locomotion in worms fed *Lactococcus* strains was mediated by dietary restriction (DR), we measured the body size and brood size, which are usually reduced under DR conditions in *C. elegans*. The body size of the nematodes was significantly decreased with LL100933 or LL12007 feeding when compared to that in the OP50-fed animals (Figure 4A,B). In contrast, the brood size of the worms fed a combination of OP50 and LL100933 or LL12007 was not reduced when compared to that of the worms fed OP50 alone (Figure 5A); however, the brood size of the worms fed LL100933 or LL12007 alone was smaller than that of the worms fed OP50 alone (Figure 5B). These results suggest that the enhanced locomotion caused by *Lactococcus* feeding was not solely due to DR.

### 3.4. Lipid Accumulation

**Figure 6:**
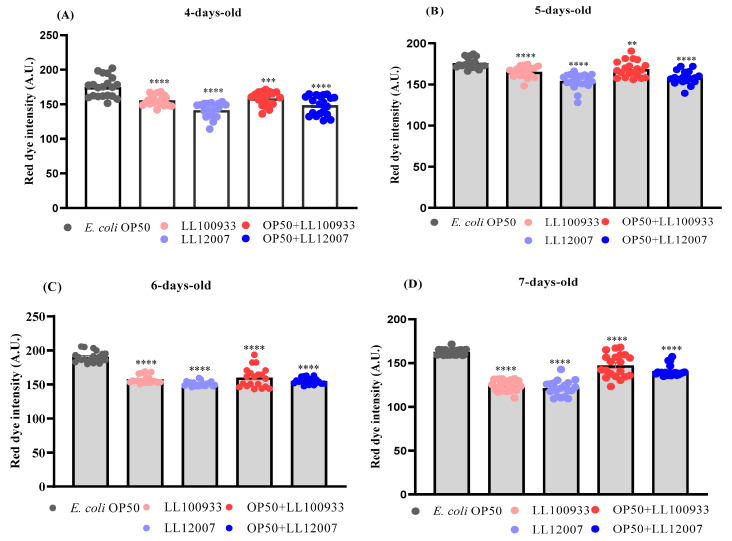
Quantification of red dye intensity in the (**A**) 4-day-old (*n* = 20); (**B**) 5-day-old (*n* = 20); (**C**) 6-day-old; and (**D**) 7-day-old worms using Oil red O staining based on the projection area of the worm body. Worms of the mixed-fed and sole-fed groups showed statistically lower lipid accumulation (lower red dye intensity) inside the body compared with that in control-fed worms. Error bars represent the standard errors. Asterisks indicate statistically significant differences (**, *p* ≤ 0.005; ***, *p* ≤ 0.0005; ****, *p* ≤ 0.0001) from the control worms (OP50-fed) using Student’s *t*-test.

**Figure 7:**
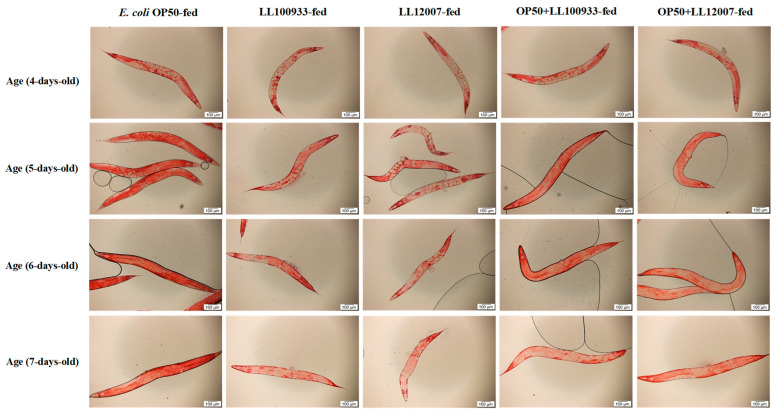
Microscopic illustration to measure the red dye intensity in control-fed (OP50), mixed-fed (OP50 + LL100933 or OP50 + LL12007), and sole-fed (LL100933 or LL12007) worms. The higher dye intensity indicates higher lipid accumulation in the worms. ImageJ software was used to measure the red dye intensity absorbed by the worm’s body. The scale bar indicates 100 μm. Magnification, ×10.

Aging disrupts lipid metabolism regulation in various organisms [30]. Therefore, we assessed whether the administration of LL100933 or LL12007 affected the accumulation of lipid droplets during aging. Oil Red O staining was used to evaluate the lipid accumulation in worms from different feeding groups: the control group, which was fed OP50, and the test-fed groups, including the mixed-fed groups (OP50 + LL100933 and OP50 + LL12007) and the sole-fed groups (LL100933 and LL12007). This experiment was conducted using worms between four and seven days of age. The lipid accumulation in the worms of the test-fed group was significantly lower (*p* < 0.0001) than that in the control-fed group at all experimental ages (Figure 6). Figure 7 illustrates the presence of worms at all experimental ages through microscopic visualization using Oil Red O staining.

### 3.5. Bacterial Choice Assay

**Figure 8:**
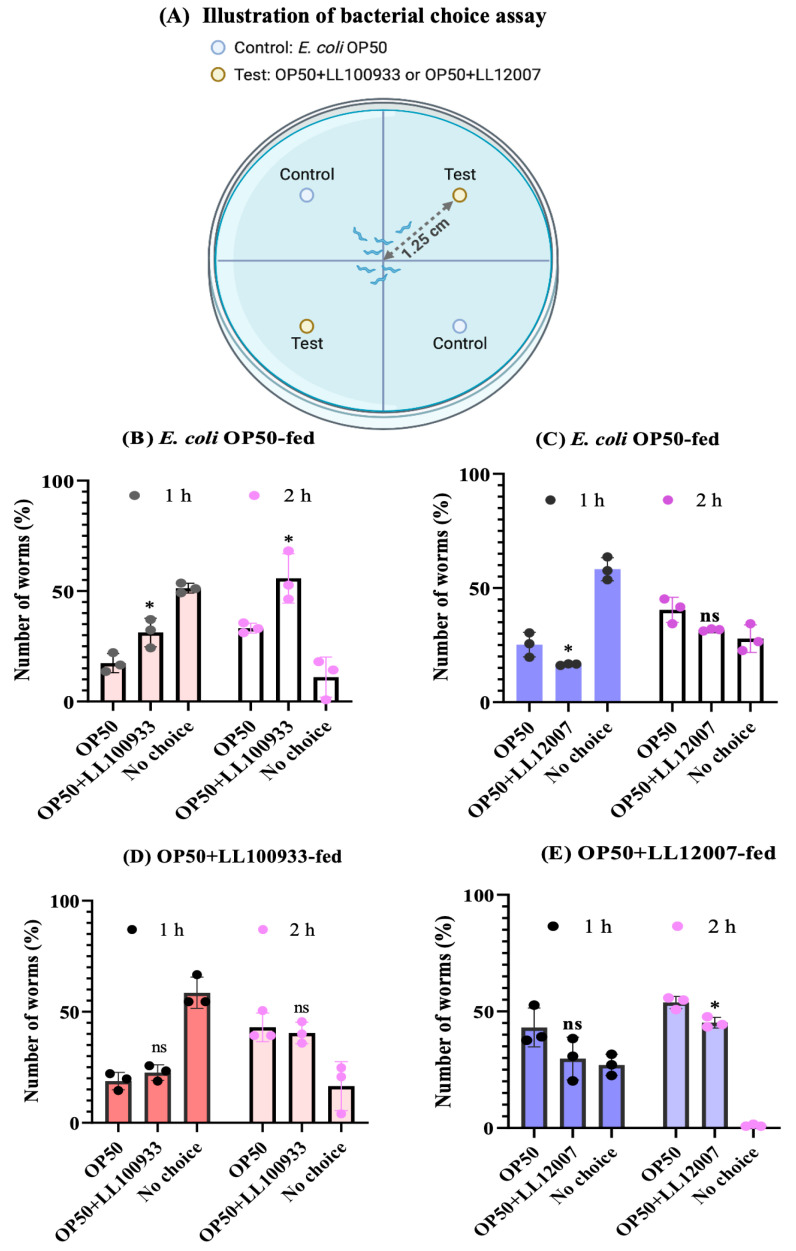
Bacterial choice assay of *C. elegans* N2. Figures (**A**) illustrates the bacterial choices of worms fed *E. coli* OP50 (**B**,**C**), OP50 + LL100933 (**D**), and OP50 + LL 12007 (**E**). Young adult (day 1) worms were fed OP50, OP50 + LL 100933, and OP50 + LL 12007 for 2 consecutive days; Day 3 aged worms were assayed for bacterial choice using the control feed (OP50) and test feed (OP50 + LL 100933 or OP50 + LL 12007) on mNGM plates (5 cm). The percentage of worms (means ± standard error) moving to the specified bacterial lawns was investigated at different time intervals (1 h and 2 h). No choice, the percentage of worms outside the bacterial lawns. Asterisks indicate statistically significant differences (*, *p* ≤ 0.05) among the worms. Three independent replicates were used for this assay and the data were analyzed using Student’s *t*-test.

*Lactococcus lactis* and *E. coli* OP50 belong to distinct bacterial genera. Studies have indicated that nematodes show a predilection when their usual food source, *Escherichia coli* OP50 (*E. coli* OP50), is replaced with alternative bacterial species [31]. Initially, we employed choice assays to investigate whether the worms exhibited a predilection for OP50 over OP50 + LL100933 or OP50 + LL12007, as shown in Figure 8A. The results indicated varying worm populations on each plate, implying a discernible preference among *C. elegans* for OP50, OP50 + LL100933, and OP50 + LL12007. Regarding the worms fed OP50, there was a notable increase in the number of worms that migrated to the OP50 + LL100933 lawn (Figure 8B), whereas the number of worms that migrated to the OP50 + LL12007 lawn was reduced after 1 h and 2 h (Figure 8C). The worms fed OP50 + LL100933 exhibited a similar preference to those fed OP50 and OP50 + LL100933 (Figure 8D). The worms fed OP50 + LL100933 showed a slightly lower preference for OP50 + LL12007 when compared to OP50 only in the 2 h assays (Figure 8E). These findings suggested that the worms displayed a preference for the OP50 + LL100933 combination over OP50 alone; however, their preference for the OP50 + LL12007 bacterial lawn was less significant. Taken together, these results suggest that a mixture of OP50 and either LL100933 or LL12007 may serve as a suitable diet for *C. elegans*.

### 3.6. Regulation of Genes with Lactococcus Feeding

**Figure 9:**
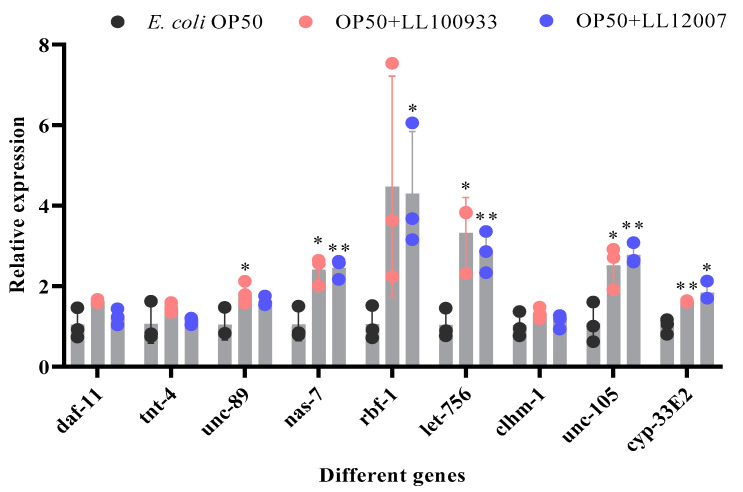
Analysis of relative mRNA expression in OP50 + LL100933-fed and OP50 + LL 12007-fed worms compared with that in control (OP50-fed) worms using real-time PCR. The figure indicates the different upregulated mRNA expressions (means ± SEM) and asterisks indicate statistically significant differences between the control (OP50-fed) and test (OP50 + LL100933-fed or OP50 + LL12007-fed) groups; *, *p* ≤ 0.05; **, *p* ≤ 0.005. Student’s *t*-test was used to measure statistical differences and three (*n* = 3) biological replicates were used.

RNA sequencing was performed to identify the genes that were influenced by administering a mixed culture of OP50 + LL100933 and OP50 + LL12007. Compared to the control-fed group, the group fed OP + LL100933 exhibited an increase in the expression of 405 genes (Table S1), while the OP + LL12007 group exhibited 1023 upregulated genes (Table S2), with a P-adjusted value of less than 0.05. Supplementary Table S3 shows the genes that were downregulated in the worms fed OP50 + LL100933, whereas Table S4 shows the downregulated genes in the worms fed OP50 + LL12007. The Gene Ontology (GO) terms that showed notable enrichment were determined for the upregulated and downregulated genes within the OP50 + LL100933- (Tables S5 and S7) and OP50 + LL12007-fed (Tables S6 and S8) groups. Among the genes whose expression was elevated in both groups (OP50 + LL100933-fed and OP50 + LL12007-fed), the Gene Ontology terms for biological processes (BP) such as the “muscle system process”, “pharyngeal pumping”, and “regulation of locomotion”, which are closely associated with locomotion, were significantly enriched (Tables S5 and S6). These terms encompass the genes responsible for encoding proteins such as the troponin complex, zinc metalloproteinase, calcium homeostasis modulator protein, degenerin-like protein, and muscle M-line assembly protein. Using real-time PCR, we confirmed that the OP50 + LL100933-fed group had higher mRNA expression levels of *unc-89* and *nas-7* than the control-fed group (Fold change: 1.831 ± 0.156; 2.403 ± 0.200, respectively) (Figure 9). The OP50 + LL12007-fed group exhibited increased mRNA expression of *rbf-1*, *let-756*, *unc-105*, and *cyp-33E2* (Fold change: 4.297 ± 0.892; 2.851 ± 0.295; 2.774 ± 0.154; and 1.837 ± 0.145, respectively) (Figure 9).

## 4. Discussion

Several previous studies have indicated that probiotics can be advantageous to their hosts by enhancing the intestinal microecological equilibrium [32,33], regulating the immune system [34,35], and adhering to the intestinal tract [36]. 

The findings of this study indicated that the *Lactococcus lactis* subsp. *lactis* strains (NBRC 100933 and NBRC 12007) did not have a notable impact on prolonging the lifespan of *C. elegans*. However, these strains had positive effects on the locomotor ability of *C. elegans*. The improved movement was demonstrated to occur via the modulation of the p38 MAPK and *skn-1* signaling pathways.

Aging is also associated with reduced muscle function and increased lipid accumulation [37]. Our results indicated that the administration of LL100933 and LL12007 improved locomotor function, increased body bending frequency, and reduced lipid buildup, suggesting that LL100933 and LL12007 improved the health span of the hosts. This aligns with earlier research findings indicating that *Weissella* [38], Probio-M9 [39], and *Lactococcus cremoris* subsp. *cremoris* [15] similarly enhance mobility and body bending and reduce lipid buildup in aging worms. The reduced lipid build-up in elderly worms could be attributed to the presence of peptides or amino acids released by bacteria with anti-obesity properties [40,41].

The body sizes of the worms in both the mixed- and sole-fed groups exhibited varying reductions at different experimental ages, as depicted in Figure 4A,B. The mixed-fed worms (OP50 + LL100933 and OP50 + LL12007) exhibited an increase in the brood size (Figure 5A), whereas the sole-fed worms (LL100933 and LL12007) showed a decrease in the brood size (Figure 5B). These findings are consistent with those of a prior investigation [42] in which CBM 588 exhibited similar effects on the reproductive rate and body size of worms. Similar to CBM 588, *Lactococcus lactis* subsp. *lactis* (LL100933 and LL12007) is believed to have the potential to enhance locomotion, partially via a DR (also known as caloric restriction, CR) mechanism. According to earlier reports, the lifespan of worms is prolonged through CR, involving the activation of DAF-16 [43] and increasing the longevity of worms through caloric restriction while preserving their fertility presents a challenging endeavor [44]. Notably, in this study, the impact of LL100933 and LL12007 was observed to be DAF-16-independent, suggesting that CR is not involved in the improved locomotion of *Lactococcus* strains.

**Figure 10:**
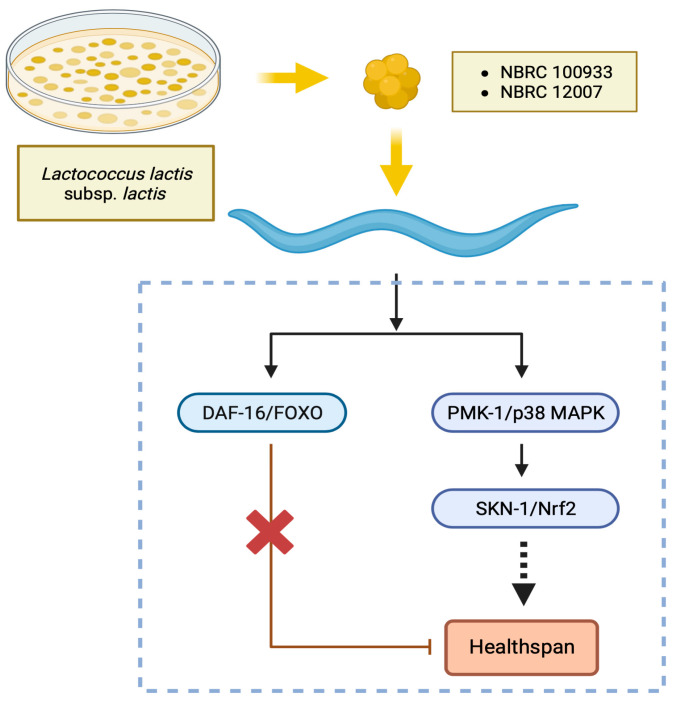
The expected mechanism for improving health span with LL100933 and LL12007 is believed to involve the enhancement of locomotor activity in *C. elegans*. This enhancement is likely achieved through modulation of the PMK-1/p38 MAPK and SKN-1/Nrf2 transcription factors, whereas DAF-16 does not seem to play a role in enhancing the motion capability of *C. elegans* when exposed to LL.

PMK-1 and SKN-1 are involved in the pathways related to stress resilience, immune system activation, metabolic processes, and detoxification [45]. The center of attention in the insulin/insulin-like growth factor signaling pathway (IGF-1) has shifted to DAF-16, which serves as an analogous counterpart of the FOXO transcription factor in mammals. DAF-16 is triggered to bolster host resilience in situations with diminished initial signals of IGF-1, as observed in scenarios such as dietary restriction. SKN-1, a transcription factor influenced by the p38 MAPK (PMK-1) pathway, controls the activation of the genes responsible for detoxifying xenobiotics [46]. The current research demonstrated that OP50 + LL100933 and OP50 + LL12007 failed to enhance the mobility of the loss-of-function *pmk-1* and *skn-1* mutants. This suggests that PMK-1 and SKN-1 may play crucial roles in the health span improvement associated with LL100933 and LL12007 (Figure 10). Consistent with our findings, *Clostridium butyricum* MIYAIRI 588 [42] and *Bifidobacterium infantis* [18] feeding improved the health span of *C. elegans*, and these beneficial effects were partially mediated via the PMK-1 and SKN-1 signaling pathways.

Concurrently, we focused on analyzing the expression of various genes through real-time PCR. Therefore, we employed Gene Ontology (GO) enrichment to analyze upregulated genes. In the group that received OP50 + LL100933, *unc-89* and *nas-7* were upregulated. Previous studies have indicated that UNC-89 plays a crucial role in the organization of myosin filaments and functions in conjunction with ryanodine receptors and L-VGCCs within a common genetic pathway that controls the movement of *C. elegans* [47]. Conversely, *nas-7* is crucial for developing the pharyngeal muscle and regulating the pharyngeal pumping rate during development [48]. In the OP50 + LL12007-fed group, the genes *rbf-1*, *let-756*, *unc-105*, and *cyp-33E2* were significantly upregulated when compared with those in the control group fed OP50. Previous findings have indicated the significant involvement of rabphillin-1 (RBF-1) as an effector in synaptic transmission regulation by RAB-3 and RAB-27 [49]. This synaptic transmission process potentially controls the movement of *C. elegans*. The *let-756* gene functions as a fibroblast growth factor (FGF) in *C. elegans* and is implicated in a range of developmental and pathological processes [50]. Within the context of LET-756, the C-terminal region is distinct and lacks a sequence comparable to that of other fibroblast growth factors (FGF). LET-756 may thus have developed this extension and related functional abilities to compensate for the limited availability of FGF molecules within the worm lineage [51]. 

*unc-105* represents the sole *C. elegans* gene that is expected to produce degenerin (DEG)/epithelial Na^+^ channels (ENaCs). Both its expression pattern and in vivo observations have revealed that this protein is generated within body wall muscles, which are a vital component of the locomotion process [52]. CYP eicosanoids (*cyp-33E2*) are essential for controlling pharyngeal pumping and food consumption in *C. elegans* [53], potentially contributing to improvements in their locomotor activity.

The findings of the present study suggest that both *Lactococcus lactis* subsp. *lactis* strains (LL100933 and LL12007) possess the capacity to improve movement and decrease lipid buildup in *C. elegans*. It would be intriguing to investigate how these strains affect host defense and the immune responses of *C. elegans* under various stress conditions. In this study, we did not observe any distinct effect of the nisin-producing strain (LL12007) on *C. elegans*, emphasizing the need for future studies to investigate the sole effect of nisin derived from *Lactococcus lactis* subsp. *lactis*.

## 5. Conclusions

In the present study, we explored the effects of nisin-producing and non-nisin-producing strains of *Lactococcus lactis* subsp. *lactis* on the health of *C. elegans*. Both strains exhibited beneficial effects on nematode health by boosting mobility and decreasing lipid build-up. The related mechanisms seemed to depend critically on the presence of the PMK-1/p38 MAPK and SKN-1/Nrf2 transcription factors. Although the positive results observed in the worms may not necessarily ensure the effectiveness of probiotics in humans, achieving favorable outcomes remains a crucial prerequisite. 

## Data Availability

The data supporting the findings of this study are available in the Gene Expression Omnibus (GEO) (GSE243721).

## Supplementary Materials

The following supporting information can be downloaded at: https://www.mdpi.com/article/10.3390/nu15204482/s1. Table S1: Genes upregulated in worms fed with OP50 + LL100933; Table S2: Genes upregulated in worms fed with OP50 + LL12007; Table S3: Downregulated genes in worms fed with OP50 + LL100933; Table S4: Genes downregulated in worms fed with OP50 + LL12007; Table S5: Enriched gene ontology (GO) for upregulated genes in worms fed with OP50 + LL100933; Table S6: Enriched gene ontology (GO) for upregulated genes in worms fed with OP50 + LL12007; Table S7: Enriched gene ontology (GO) for downregulated genes in worms fed with OP50 + LL100933; Table S8: Enriched gene ontology (GO) for downregulated genes in worms fed with OP50 + LL12007; Table S9: Primer list; Table S10: Data of lifespan assay of *C. elegans*.

## References

[1] Deng Z., Hou K., Zhao J., Wang H. (2022). The Probiotic Properties of Lactic Acid Bacteria and Their Applications in Animal Husbandry. Curr. Microbiol..

[2] Sanders M.E. (2008). Probiotics: Definition, Sources, Selection, and Uses. Clin. Infect. Dis..

[3] Li T.T., Tian W.L., Gu C.T. (2019). Elevation of *Lactococcus lactis* subsp. *cremoris* to the Species Level as *Lactococcus cremoris* sp. nov. and Transfer of *Lactococcus lactis* subsp. *tructae* to *Lactococcus cremoris* as *Lactococcus cremoris* subsp. *tructae* comb. nov. Int. J. Syst. Evol. Microbiol..

[4] Jung M.Y., Lee C., Seo M.-J., Roh S.W., Lee S.H. (2020). Characterization of a Potential Probiotic Bacterium Lactococcus Raffinolactis WiKim0068 Isolated from Fermented Vegetable Using Genomic and in Vitro Analyses. BMC Microbiol..

[5] Belo G.A., Cordeiro B.F., Oliveira E.R., Braga M.P., Da Silva S.H., Costa B.G., Martins F.D.S., Jan G., Le Loir Y., Gala-García A. (2021). SlpB Protein Enhances the Probiotic Potential of L. Lactis NCDO 2118 in Colitis Mice Model. Front. Pharmacol..

[6] Bandyopadhyay B., Das S., Mitra P.K., Kundu A., Mandal V., Adhikary R., Mandal V., Mandal N.C. (2022). Characterization of Two New Strains of Lactococcus Lactis for Their Probiotic Efficacy over Commercial Synbiotics Consortia. Braz. J. Microbiol..

[7] Sałański P., Kowalczyk M., Bardowski J.K., Szczepankowska A.K. (2022). Health-Promoting Nature of Lactococcus Lactis IBB109 and Lactococcus Lactis IBB417 Strains Exhibiting Proliferation Inhibition and Stimulation of Interleukin-18 Expression in Colorectal Cancer Cells. Front. Microbiol..

[8] Jawan R., Abbasiliasi S., Mustafa S., Kapri M.R., Halim M., Ariff A.B. (2021). In Vitro Evaluation of Potential Probiotic Strain Lactococcus Lactis Gh1 and Its Bacteriocin-Like Inhibitory Substances for Potential Use in the Food Industry. Probiotics Antimicro. Prot..

[9] Wan X., Takala T.M., Qiao M., Saris P.E.J. (2021). Complete Genome Sequence of Nisin-Producing *Lactococcus lactis* subsp. *Lactis* N8. Microbiol. Resour. Announc..

[10] Zhao G., Liu J., Zhao J., Dorau R., Jensen P.R., Solem C. (2021). Efficient Production of Nisin A from Low-Value Dairy Side Streams Using a Nonengineered Dairy *Lactococcus lactis* Strain with Low Lactate Dehydrogenase Activity. J. Agric. Food Chem..

[11] Finch C.E., Ruvkun G. (2001). The Genetics of Aging. Annu. Rev. Genom. Hum. Genet..

[12] Roselli M., Schifano E., Guantario B., Zinno P., Uccelletti D., Devirgiliis C. (2019). *Caenorhabditis elegans* and Probiotics Interactions from a Prolongevity Perspective. Int. J. Mol. Sci..

[13] Poupet C., Chassard C., Nivoliez A., Bornes S. (2020). *Caenorhabditis elegans*, a Host to Investigate the Probiotic Properties of Beneficial Microorganisms. Front. Nutr..

[14] Petersen C., Dierking K., Johnke J., Schulenburg H. (2022). Isolation and Characterization of the Natural Microbiota of the Model Nematode *Caenorhabditis elegans*. JoVE J. Vis. Exp..

[15] Komura T., Takemoto A., Kosaka H., Suzuki T., Nishikawa Y. (2022). Prolonged Lifespan, Improved Perception, and Enhanced Host Defense of *Caenorhabditis elegans* by *Lactococcus cremoris* subsp. Cremoris. Microbiol. Spectr..

[16] Brenner S. (1974). The Genetics of *Caenorhabditis elegans*. Genetics.

[17] Wu D., Rea S., Yashin A., Johnson T. (2006). Visualizing Hidden Heterogeneity in Isogenic Populations of *C. elegans*. Exp. Gerontol..

[18] Komura T., Ikeda T., Yasui C., Saeki S., Nishikawa Y. (2013). Mechanism Underlying Prolongevity Induced by Bifidobacteria in *Caenorhabditis elegans*. Biogerontology.

[19] Gruber J., Ng L.F., Fong S., Wong Y.T., Koh S.A., Chen C.-B., Shui G., Cheong W.F., Schaffer S., Wenk M.R. (2011). Mitochondrial Changes in Ageing *Caenorhabditis elegans*—What Do We Learn from Superoxide Dismutase Knockouts?. PLoS ONE.

[20] Pompa L., Montanari A., Tomassini A., Bianchi M.M., Aureli W., Miccheli A., Uccelletti D., Schifano E. (2023). In Vitro Probiotic Properties and in Vivo Anti-Ageing Effects of Lactoplantibacillus Plantarum PFA2018AU Strain Isolated from Carrots on *Caenorhabditis elegans*. Microorganisms.

[21] Soete G., Betist M.C., Korswagen H.C. (2007). Regulation of *Caenorhabditis elegans* Body Size and Male Tail Development by the Novel Gene Lon-8. BMC Dev. Biol..

[22] Gusarov I., Pani B., Gautier L., Smolentseva O., Eremina S., Shamovsky I., Katkova-Zhukotskaya O., Mironov A., Nudler E. (2017). Glycogen Controls *Caenorhabditis elegans* Lifespan and Resistance to Oxidative Stress. Nat. Commun..

[23] Chow Y.-L., Sato F. (2013). Screening of Isoquinoline Alkaloids for Potent Lipid Metabolism Modulation with *Caenorhabditis elegans*. Biosci. Biotechnol. Biochem..

[24] Yen K., Le T.T., Bansal A., Narasimhan S.D., Cheng J.-X., Tissenbaum H.A. (2010). A Comparative Study of Fat Storage Quantitation in Nematode *Caenorhabditis elegans* Using Label and Label-Free Methods. PLoS ONE.

[25] Bolger A.M., Lohse M., Usadel B. (2014). Trimmomatic: A Flexible Trimmer for Illumina Sequence Data. Bioinformatics.

[26] Dobin A., Davis C.A., Schlesinger F., Drenkow J., Zaleski C., Jha S., Batut P., Chaisson M., Gingeras T.R. (2013). STAR: Ultrafast Universal RNA-Seq Aligner. Bioinformatics.

[27] Liao Y., Smyth G.K., Shi W. (2014). featureCounts: An Efficient General Purpose Program for Assigning Sequence Reads to Genomic Features. Bioinformatics.

[28] Anders S., Huber W. (2010). Differential Expression Analysis for Sequence Count Data. Nat. Preced..

[29] Livak K.J., Schmittgen T.D. (2001). Analysis of Relative Gene Expression Data Using Real-Time Quantitative PCR and the 2^−ΔΔCT^ Method. Methods.

[30] Rubio-Tomás T., Tavernarakis N. (2022). Lipid Metabolism and Ageing in *Caenorhabditis elegans*: A Complex Interplay. Biogerontology.

[31] Abada E.A., Sung H., Dwivedi M., Park B.-J., Lee S.-K., Ahnn J. (2009). *C. elegans* Behavior of Preference Choice on Bacterial Food. Mol. Cells.

[32] Malaguarnera G., Leggio F., Vacante M., Motta M., Giordano M., Biondi A., Basile F., Mastrojeni S., Mistretta A., Malaguarnera M. (2012). Probiotics in the Gastrointestinal Diseases of the Elderly. J. Nutr. Health Aging.

[33] Azad M.A.K., Sarker M., Li T., Yin J. (2018). Probiotic Species in the Modulation of Gut Microbiota: An Overview. BioMed Res. Int..

[34] Komura T., Ikeda T., Hoshino K., Shibamura A., Nishikawa Y., Mylonakis E., Ausubel F.M., Gilmore M., Casadevall A. (2012). Caenorhabditis elegans as an Alternative Model to Study Senescence of Host Defense and the Prevention by Immunonutrition. Recent Advances on Model Hosts.

[35] Park M.R., Ryu S., Maburutse B.E., Oh N.S., Kim S.H., Oh S., Jeong S.-Y., Jeong D.-Y., Oh S., Kim Y. (2018). Probiotic Lactobacillus Fermentum Strain JDFM216 Stimulates the Longevity and Immune Response of *Caenorhabditis elegans* through a Nuclear Hormone Receptor. Sci. Rep..

[36] Oelschlaeger T.A. (2010). Mechanisms of Probiotic Actions—A Review. Int. J. Med. Microbiol..

[37] Pincus Z., Slack F.J. (2010). Developmental Biomarkers of Aging in *Caenorhabditis elegans*. Dev. Dyn..

[38] Lee J., Kwon G., Lim Y.-H. (2015). Elucidating the Mechanism of Weissella-Dependent Lifespan Extension in *Caenorhabditis elegans*. Sci. Rep..

[39] Zhang J., Zhao Y., Sun Z., Sun T. (2022). Lacticaseibacillus Rhamnosus Probio-M9 Extends the Lifespan of *Caenorhabditis elegans*. Commun. Biol..

[40] Brooks K.K., Liang B., Watts J.L. (2009). The Influence of Bacterial Diet on Fat Storage in *C. elegans*. PLoS ONE.

[41] Yoon S., Cho H., Nam Y., Park M., Lim A., Kim J.-H., Park J., Kim W. (2022). Multifunctional Probiotic and Functional Properties of *Lactiplantibacillus plantarum* LRCC5314, Isolated from Kimchi. J. Microbiol. Biotechnol..

[42] Kato M., Hamazaki Y., Sun S., Nishikawa Y., Kage-Nakadai E. (2018). Clostridium Butyricum MIYAIRI 588 Increases the Lifespan and Multiple-Stress Resistance of *Caenorhabditis elegans*. Nutrients.

[43] Berdichevsky A., Viswanathan M., Horvitz H.R., Guarente L. (2006). *C. elegans* SIR-2.1 Interacts with 14-3-3 Proteins to Activate DAF-16 and Extend Life Span. Cell.

[44] Greer E.L., Brunet A. (2009). Different Dietary Restriction Regimens Extend Lifespan by Both Independent and Overlapping Genetic Pathways in *C. elegans*. Aging Cell.

[45] Blackwell T.K., Steinbaugh M.J., Hourihan J.M., Ewald C.Y., Isik M. (2015). SKN-1/Nrf, Stress Responses, and Aging in *Caenorhabditis elegans*. Free Radic. Biol. Med..

[46] Choe K.P., Leung C.K., Miyamoto M.M. (2012). Unique Structure and Regulation of the Nematode Detoxification Gene Regulator, SKN-1: Implications to Understanding and Controlling Drug Resistance. Drug Metab. Rev..

[47] Spooner P.M., Bonner J., Maricq A.V., Benian G.M., Norman K.R. (2012). Large Isoforms of UNC-89 (Obscurin) Are Required for Muscle Cell Architecture and Optimal Calcium Release in *Caenorhabditis elegans*. PLoS ONE.

[48] Park J.-O., Pan J., Möhrlen F., Schupp M.-O., Johnsen R., Baillie D.L., Zapf R., Moerman D.G., Hutter H. (2010). Characterization of the Astacin Family of Metalloproteases in *C. elegans*. BMC Dev. Biol..

[49] Mahoney T.R., Liu Q., Itoh T., Luo S., Hadwiger G., Vincent R., Wang Z.-W., Fukuda M., Nonet M.L. (2006). Regulation of Synaptic Transmission by RAB-3 and RAB-27 in *Caenorhabditis elegans*. Mol. Biol. Cell.

[50] Roubin R., Naert K., Popovici C., Vatcher G., Coulier F., Thierry-Mieg J., Pontarotti P., Birnbaum D., Baillie D., Thierry-Mieg D. (1999). Let-756, a *C. elegans* Fgf Essential for Worm Development. Oncogene.

[51] Popovici C., Fallet M., Marguet D., Birnbaum D., Roubin R. (2006). Intracellular Trafficking of LET-756, a Fibroblast Growth Factor of *C. elegans*, Is Controlled by a Balance of Export and Nuclear Signals. Exp. Cell Res..

[52] Jospin M., Mariol M.-C., Segalat L., Allard B. (2004). Patch Clamp Study of the UNC-105 Degenerin and Its Interaction with the LET-2 Collagen in *Caenorhabditis elegans* Muscle: UNC-105 Degenerin in *C. elegans* Muscle. J. Physiol..

[53] Zhou Y., Falck J.R., Rothe M., Schunck W.-H., Menzel R. (2015). Role of CYP Eicosanoids in the Regulation of Pharyngeal Pumping and Food Uptake in *Caenorhabditis elegans*. J. Lipid Res..

