# Supplementary material for: Improvement of Locomotion Caused by Lactococcus lactis subsp. lactis in the Model Organism Caenorhabditis elegans
Source: Nutrients. 2023 Oct 23;15(20):4482. doi: 10.3390/nu15204482 (PMC10610199; doi:10.3390/nu15204482)
Supplement: Supplementary file 1 [file nutrients-15-04482-s001.zip › Ali_Suppl_Nutrients2023/Table S10.docx]

**Table S10: Lifespan assay of *C. elegans*.**

| **Genotype** | **Trials** | **Treatment** | **MLS (days±SEM)** | **N** | **Censored** |
| --- | --- | --- | --- | --- | --- |
| Wild type (N2) | 1 | *E. coli* OP50 | 17.37±0.62 | 61 | 9 |
|  |  | OP50+LL 100933 | 17.25±0.79 | 56 | 14 |
|  |  | OP50+LL 12007 | 17.81±0.60 | 62 | 8 |
|  | 2 | *E. coli* OP50 | 16.60±0.49 | 62 | 8 |
|  |  | OP50+LL 100933 | 16.14±0.51 | 53 | 17 |
|  |  | OP50+LL 12007 | 16.53±0.47 | 61 | 9 |
|  | CD | *E. coli* OP50 | 16.98±0.39 | 123 | 17 |
|  |  | OP50+LL 100933 | 17.13±0.48 | 109 | 31 |
|  |  | OP50+LL 12007 | 17.18±0.38 | 123 | 17 |
| *daf-16* (*mu86*) | 1 | *E. coli* OP50 | 13.29±0.39 | 58 | 12 |
|  |  | OP50+LL 100933 | 16.39±0.52 | 58 | 12 |
|  |  | OP50+LL 12007 | 14.19±0.52 | 60 | 10 |
|  | 2 | *E. coli* OP50 | 13.87±0.50 | 61 | 9 |
|  |  | OP50+LL 100933 | 16.48±0.56 | 59 | 11 |
|  |  | OP50+LL 12007 | 16.82±0.52 | 58 | 12 |
|  | CD | *E. coli* OP50 | 13.59±0.32 | 119 | 21 |
|  |  | OP50+LL 100933 | 16.44±0.38 | 117 | 23 |
|  |  | OP50+LL 12007 | 15.48±0.37 | 118 | 22 |
| *skn-1* (*ok2315*) | 1 | *E. coli* OP50 | 15.03±0.65 | 58 | 12 |
|  |  | OP50+LL 100933 | 12.57±0.56 | 52 | 18 |
|  |  | OP50+LL 12007 | 12.08±0.53 | 55 | 15 |
|  | 2 | *E. coli* OP50 | 13.71±0.60 | 62 | 8 |
|  |  | OP50+LL 100933 | 13.68±0.65 | 50 | 20 |
|  |  | OP50+LL 12007 | 13.19±0.63 | 53 | 17 |
|  | CD | *E. coli* OP50 | 14.35±0.44 | 120 | 20 |
|  |  | OP50+LL 100933 | 13.11±0.43 | 102 | 38 |
|  |  | OP50+LL 12007 | 12.63±0.41 | 108 | 32 |
| *pmk-1* (*km25*) | 1 | *E. coli* OP50 | 14.65±0.75 | 60 | 10 |
|  |  | OP50+LL 100933 | 14.08±0.83 | 62 | 8 |
|  |  | OP50+LL 12007 | 12.93±0.79 | 53 | 17 |
|  | 2 | *E. coli* OP50 | 16.50±0.80 | 65 | 5 |
|  |  | OP50+LL 100933 | 14.07±0.83 | 61 | 9 |
|  |  | OP50+LL 12007 | 13.86±0.79 | 57 | 13 |
|  | CD | *E. coli* OP50 | 15.61±0.43 | 125 | 15 |
|  |  | OP50+LL 100933 | 14.07±0.42 | 123 | 17 |
|  |  | OP50+LL 12007 | 13.41±0.46 | 110 | 30 |
